# Enzyme Feed Additive with Arazyme Improve Growth Performance, Meat Quality, and Gut Microbiome of Pigs

**DOI:** 10.3390/ani13030423

**Published:** 2023-01-26

**Authors:** Jong-Hoon Kim, Bon-Hwan Ku, Gwang-Pyo Ko, Man-Jong Kang, Kwang-Hee Son, Mi-Ae Bang, Ho-Yong Park

**Affiliations:** 1 Microbiome Convergence Research Center, Korea Research Institute of Bioscience and Biotechnology, Daejeon 34141, Republic of Korea; 2 Insect Biotech Co., Ltd., Daejeon 34054, Republic of Korea; 3 Department of Animal Science, Chonnam National University, Gwangju 61186, Republic of Korea; 4 Department of Food Industry Research Center, Jeonnam Bioindustry Foundation, Naju 58275, Republic of Korea

**Keywords:** arazyme, feed additive, meat quality, microbial community, pig

## Abstract

**Simple Summary:**

Supplementation of exogenous enzymes in a diet, particularly protease as a part of an enzyme mixture, is widely used with the expectation that it will enhance the efficiency of nutrient utilization by neutralizing anti-nutritional factors and increasing nutrient digestibility and, thereby, improving growth performance. In our study, dietary supplementation of exogenous enzymes derived from invertebrate symbiotic bacteria affected the growth performance, meat quality, and gut microbiota of pigs. The results of this study suggest that applying exogenous enzymes derived from invertebrate symbiotic bacteria enhances animal performance and can be used as a theoretical basis for further studies.

**Abstract:**

The supplementation of pig diets with exogenous enzymes is widely used with the expectation that it will improve the efficiency of nutrient utilization, thereby, improving growth performance. This study aims to evaluate the effects of a 0.1% (*v*/*v*) multi-enzyme (a mixture of arazyme (2,500,000 Unit/kg), xylanase (200,000 Unit/kg) and mannanase (200,000 Unit/kg)) supplementation derived from invertebrate symbiotic bacteria on pig performance. Here, 256 growing pigs were assigned to control and treatment groups, respectively. The treatment group exhibited a significantly reduced average slaughter age; the final body weight and average daily gain increased compared with that of the control group. In the treatment group, the longissimus muscle showed a remarkable decrease in cooking loss, shear force, and color values with increased essential and non-essential amino acid concentrations. Furthermore, the concentrations of mono- and polyunsaturated fatty acids in the treatment group increased. Feed additive supplementation increased the family of Ruminococcaceae and genera *Lactobacillus*, *Limosilactobacillus*, *Turicibacter*, and *Oscillibacter*, which play a positive role in the host physiology and health. Predicted metabolic pathway analysis confirmed that operational taxonomic units and predicted amino acid biosynthesis pathways were strongly associated. The results suggest that applying exogenous enzymes derived from invertebrate symbiotic bacteria enhances animal performance.

## 1. Introduction

Exogenous enzyme supplementation of pig diets was stimulated recently in the pig industry as a strategy to enhance the growth performance of growing–finishing pigs. Various exogenous enzymes, such as proteases, carbohydrases, and phytases, are commercially supplemented alone or as part of a multi-enzyme combination and have shown positive effects on feed utilization and animal performance in pigs. These substrate-specific enzymes convert the indigestible components of feed ingredients into substrates that the pig can digest. Many studies reported that the supplementation of a multi-enzyme has a more positive effect on pigs due to the synergistic interaction between enzymes [1]. Non-starch polysaccharide (NSPs)-degrading enzymes such as xylanase, mannanase, and glucanase hydrolyze plant-cell-wall components of the diet and assist in the release of nutritional constituents such as proteins, lipids, starch, and minerals trapped within the cell-wall matrix [2]. Additionally, protease complements the proteolytic activity by hydrolyzing the proteins resistant to endogenous secretory enzymes, improving the digestibility of amino acids, disrupting the feed structure, liberating fat and starch, and inactivating the anti-nutritional factors present in the diet [3]. Therefore, adding exogenous dietary enzymes may improve the digestibility and utilization of nutrients, benefiting host performance and health. Hence, the swine industry is looking for solutions to improve the availability of nutrients in pig diets.

Pig intestines are colonized by diverse microbes that contribute to several biological functions and play an important role in the physiology and health of the host. It has significant effects in various aspects, such as suppressing pathogenic infections, synthesizing essential vitamins and amino acids, regulating fat metabolism, and forming immune systems [4]. Undigested nutrition can eventually become a source of pathogenic bacteria in the large intestines, forming amino-acid-derived metabolites that are toxic to the intestinal epithelium of pigs. Therefore, the hydrolysis of NSPs and proteins is crucial for a balanced intestinal microbiota and for improving the health and productivity of the host. However, studies determining the effect of dietary supplementation of enzymes on pigs’ microbial composition and host–microbiome interactions are limited.

Invertebrates have developed symbiotic interactions with different microorganisms that secrete most of the metabolism-specific digestive enzymes to overcome nutritional limitations [5]. The study of invertebrate gut-associated symbionts currently represents multidimensional industrial capabilities of biotechnologically active enzymes such as amylase, cellulase, protease, lipase, xylanase, and pectinase. Additionally, arazyme is a 51.5 kDa metalloprotease purified from *Serratia proteamaculans* HY-3, a Gram-negative symbiotic bacterium of the spider *Nephila clavata* [6]. Purified arazyme showed high relative proteolytic activities and reduced the viscosity and ammonia concentration of intestinal contents, reflecting a significant feed enzyme effect [7]. However, the experimental evidence of the effects of exogenous enzymes derived from invertebrate symbionts as feed additives for pigs is limited. Therefore, this study focused on evaluating the effects of a multi-enzyme supplement with arazyme on meat quality, gut microbiota, and host-microbiome interactions in pigs.

## 2. Materials and Methods

### 2.1. Experimental Diets and Feed Additives

This study used a commercial product (Special One^®^, TS Corporation, Seoul, Republic of Korea) as the diet for the pigs. The ingredient and chemical composition of the experimental diets are listed in Table 1 and Table 2. The energy supplementation quantity of the diets was based on the manufacturer’s recommendations. Arazyme was the main ingredient of feed additives in this study. The mannan-degrading enzyme (Mannanase, ManK, 34.9 kDa) was purified from *Cellulosimicrobium* sp. HY-13, a gut bacterium of *Eisenia fetida* [8]. The xylanase (XynA, 19.9 kDa) was purified from *Paenibacillus* sp. HY-8, a gut bacterium of *Moechotypa diphysis* [9]. The tested feed additives contained a mixture of arazyme (2,500,000 Units/kg) and synergetic enzymes (xylanase (200,000 Units/kg) and mannanase (200,000 Units/kg)) in a 12.5:1:1 ratio. It was provided by InsectBiotech Co., Ltd. (Daejeon, Republic of Korea).

### 2.2. Animals and Experimental Design

A total of 256 growing pigs [Duroc (Landrace × Yorkshire)] with an initial average body weight of 37.90 ± 3.88 kg were used as the control group, and 256 pigs with an initial average body weight of 37.57 ± 3.24 kg were assigned to the treatment group. All animals used were male. There were eight replicate pens with 32 pigs per replicate in each experimental group (4.0 m × 7.4 m; approximately 0.93 m^2^ per pig). The control group was fed a basal diet, and the treatment group was fed the basal diet supplemented with 0.1% enzyme mixture (*v*/*v*) ad libitum for the entire experimental period. Pigs were housed in an environmentally controlled system. Each pen was equipped with a stainless-steel self-feeder and a low-pressure nipple drinker that provided feed and water throughout the experimental period. The experiment for each group was complete when the average body weight of pigs per group was over 110 kg, according to Korea Institute for Animal Products Quality Evaluation. The initial and final body weights of eight pigs in each group were recorded to calculate the average daily gain (ADG). Feed intake was measured to determine feed efficiency by calculating the gain:feed ratio.

### 2.3. Sample Preparation

At the end of the experiment, eight pigs per group with similar body weights to average body weight (110.24 ± 0.64 kg, one pig per pen) were selected and rectal swabs were conducted for subsequent microbial 16S rRNA sequencing. A total of selected 16 pigs (eight pigs per group) were transported to a local commercial slaughter plant (Jeonnam, Republic of Korea). The pigs were slaughtered by exsanguination after electrical stunning and placed in a dehairer at 65 °C for 5 min. The carcass was longitudinally and symmetrically split after removing the head, feet, tail, and viscera, except for the suet and kidneys. The longissimus muscle samples in each selected carcass were immediately removed for meat quality determination.

### 2.4. Meat Quality Determination

The meat qualities were determined 24 h after slaughter. The pH values of the longissimus muscle were measured using a digital pH meter with a penetrating metal probe (Thermo Orion 555A, Thermo Fisher Scientific Inc., Waltham, MA, USA). The color measurement was performed on longissimus muscle cubes (4 cm × 4 cm × 4 cm). The color parameters lightness (L*), redness (a*), and yellowness (b*) were determined at three different sites of the samples using a spectrophotometer (ColorTouch ^®^ II; Technidyne Co., New Albany, IN, USA). The average values of each parameter were recorded. The longissimus muscle was cut into cubes (2 × 2 × 2 cm), and the cooking loss was determined. The initial weight of each longissimus muscle cube was recorded before cooking to investigate the cooking loss. The sample was incubated in a water bath at 75 °C for 1 h and the final weight was recorded after cooling to 28 °C. The cooking loss percentage was obtained by determining the weight change of the sample before and after cooking. After determining cooking loss, the same samples were placed at the blades (round adapter) of a rheometer (Compac-100II, Sun Scientific Co., Tokyo, Japan). The weight was calibrated with a 2 kg weight, and the return speed was set at 2 mm/s. The shear force values were recorded from each strip. Drip loss was determined using the method described by Logan et al. [10]. Thiobarbituric acid reactive substances (TBARS) were determined using the method described by Zeb et al. [11] with minor modifications. Briefly, 1 g of longissimus muscle was homogenized in 5 mL of acetic acid (50%) and 50 μL of butylated hydroxytoluene and centrifuged at 3000× *g* at 4 °C for 10 min. Next, 1 mL of supernatant was transferred to a 15 mL test tube, and 1 mL of thiobarbituric acid/trichloroacetic acid solution (4 mM) was added. The mixture was incubated in a 90 °C water bath for 1 h and cooled to 28 °C. The mixture’s absorbance was measured at 531 nm using a spectrophotometer (DU 730^®^ Life Science UV/Vis spectrophotometer; Beckman Coulter, Brea, CA, USA). The concentration of TBARS in malondialdehyde (MDA) was calculated as mg kg^−1^ of the longissimus muscle sample. To investigate the antioxidant capacity of longissimus muscle samples, 50 mg of longissimus muscle was homogenized in 1 mL of distilled water. The 1,1-diphenyl-2-picrylhydrazyl (DPPH) radical scavenging activity of longissimus muscle was determined as described by Bland-Williams et al. [12], with minor modifications. A mixture of 10 μL of sample and 190 μL of 0.1 mM DPPH solution (Wako, Osaka, Japan) was incubated in the dark for 10 min, and the absorbance was measured at 517 nm. The DPPH radical scavenging activity was calculated as follows: DPPH radical scavenging activity (%) = [1 − (Abs_sample_/Abs_blank_)] × 100. The 2,2′-azino-bis(3-ethylbenzothiazoline-6-sulfonic acid) (ABTS) radical scavenging activity was measured according to Vander Berg et al. [13] with minor modifications. The ABTS stock solution was prepared with an absorbance of 0.70 ± 0.02 at 734 nm. Then, 10 μL of the sample was added to 190 μL of ABTS solution. After a 30 min incubation at 28 °C, the absorbance was measured at 734 nm. The ABTS radical scavenging activity was determined as follows: ABTS radical scavenging activity (%) = [1 − (Abs_sample_/Abs_blank_)] × 100. The experiments were performed in triplicate.

### 2.5. Measurement of Free Amino Acids and Fatty Acid Composition in Meat

The free amino acid profile of the longissimus muscle sample was measured following sample acid-hydrolysis using an automatic amino acid analyzer (L8900, Hitachi, Tokyo, Japan) according to Tian et al. [14]. The fatty acids composition of longissimus muscle was estimated using the method of O’Fallon et al. [15] with minor modifications. The fatty acids were analyzed using an Agilent 7890A Gas Chromatograph (Agilent, Santa Clara, CA, USA) under the following conditions: injector split mode with a split ratio of 10:1 and temperature 250 °C. High-purity air (350 mL/min), H2 (35 mL/min), and He (35 mL/min) were used as carrier gases. A DB-23 column (30 m × 0.25 mm × 0.25 μm; Agilent) was used for the analysis. The fatty acid composition is expressed as a percentage.

### 2.6. Fecal Microbiota Analysis

Pig fecal specimens were collected from each group, and genomic DNA was extracted from the fecal samples. Sequencing was performed by Macrogen Inc. (Seoul, Republic of Korea) to investigate the microbial composition. Briefly, the V3 and V4 hypervariable region of the 16S rRNA gene was amplified, and paired-end (2 × 300 bp) sequencing was performed using the Illumina MiSeq platform. MOTHUR software (version 1.30.2) was used to analyze the 16S rRNA sequencing data according to MiSeq SOP guidelines (https://mothur.org/wiki/miseq_sop, accessed on 18 August 2021) with a simple modification. Briefly, paired-end reads were assembled and aligned with the SILVA database (version 138). To eliminate the singletons, the split.abund MOTHUR subroutine was performed. VSEARCH was used to detect chimeric sequences and Ribosome database project (RDP, version 18) database was used to perform Taxonomic classification. Sequences other than the undesired taxa (i.e., chloroplast and mitochondria) were removed. The results were clustered using the opticlust MOTHUR subroutine to assign operational taxonomic units (OTUs). The number of reads per sample was normalized to 10,000 for downstream analyses. Alpha diversity indices for species richness (Chao and Ace) and diversity (Shannon and Invsimpson) were evaluated. Beta diversity was calculated based on Bray–Curtis dissimilarity coefficients, visualized by non-metric multidimensional scaling (NMDS) analysis. Prediction of the functional content of microbial communities was estimated using Phylogenetic Investigation of Communities by Reconstruction of Unobserved States (PICRUSt2).

### 2.7. Statistical Analysis

Results are expressed as the mean ± standard deviation. Alpha diversity indices were evaluated using analysis of variance (ANOVA). Statistical analysis was performed using the Student’s *t*-test for significant differences within the parameters and ecological indices (*p* < 0.05) and the two-sided Welch’s *t*-test for significant differences in predicted metabolic pathways (*p* < 0.01). Analysis of molecular variance (AMOVA) was used to test the significant differences in fecal microbiota. The taxonomic composition and OTUs of fecal microbiota with significant differences between groups were evaluated using the linear discriminant analysis effect size (LEfSe) based on the Kruskal–Wallis sum-rank test. Additionally, predicted significantly different metabolic pathways were investigated using ALDEx2 package in R software (https://github.com/ggloor/ALDEx2, accessed on 18 August 2021). The output from Spearman correlation analysis was filtered by the coefficient value following the user’s guide to correlation coefficients (strong positive correlation > 0.8 and strong negative correlation < −0.8).

## 3. Results

### 3.1. Growth Performance

The growth performance and carcass traits of experimental pigs are presented in Table 3. The feed additive supplementation group had a significantly reduced average slaughter age and gain:feed ratio compared with the control group. The final body weight and ADG increased (*p* < 0.05). There were no significant differences between the two groups relative to initial body weight and average daily feed intake (ADFI).

### 3.2. Meat Quality

Carcass and meat quality traits are shown in Table 4. Hot carcass weight and backfat thickness were significantly higher in the treatment group than in the control group (*p* < 0.05). The pH value, drip loss, L* value, and TBARS level showed no significant differences between the control and feed additive supplementation groups. Nevertheless, the groups differed in cooking loss, shear force, and a* and b* values (*p* < 0.05). These results showed a remarkable decrease in the treatment group compared to that in the control group. Based on the evaluation of the antioxidant capacity of longissimus muscle, the DPPH and ABTS radical scavenging activities of the feed additive group were notably higher than that in the control group (*p* < 0.05).

### 3.3. Amino Acids Profile

The amino acid profiles of the longissimus muscle are shown in Table 5. Diet supplemented with multi-enzyme greatly altered the free amino acid composition of the longissimus muscle. Compared with the control group, the treatment group showed a significant increase in total amino acids of 17.76% (*p* < 0.05). The concentration of essential amino acids (EAAs) and non-essential amino acids (NEAAs) increased by 21.47% and 14.64%, respectively, in the treatment group compared with that of the control group (*p* < 0.05). Furthermore, in the treatment group, the total amino acid content was remarkably higher than that of the control group (*p* < 0.05).

### 3.4. Fatty Acids Composition

As shown in Table 6, saturated fatty acids (SFAs) and monounsaturated fatty acids (MUFAs) were the most abundant components in the control and treatment groups. The treatment group showed a decreased concentration of SFAs, whereas the concentration of MUFAs and polyunsaturated fatty acids (PUFAs) increased relative to that of the control group (*p* < 0.05). The concentrations of C16:1n-7, C18:1n-9, C20:2, and C20:4n-6 were higher in the treatment group than in the control group (*p* < 0.05). Additionally, there was no significant difference between the control and treatment groups in terms of C20:0, C21:0, C18:2n-6, and C20:1n-9 concentrations.

### 3.5. Gut Microbiota Community

**Figure 1:**
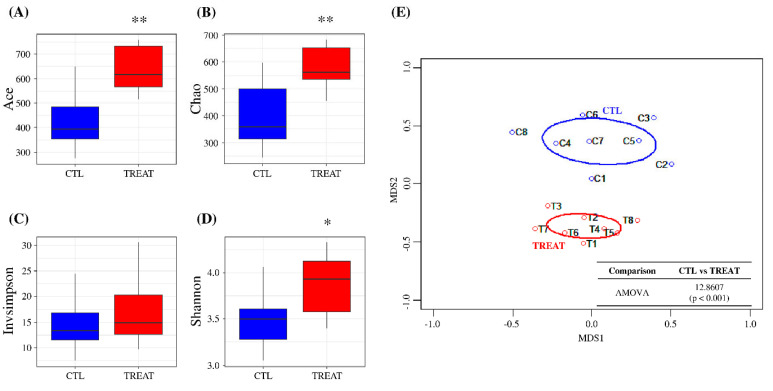
Assessment of gut microbiota using alpha- and beta-diversity indices. Ace diversity (**A**), Chao diversity (**B**), InvSimpson diversity (**C**), Shannon diversity (**D**), and non-metric multidimensional scaling (NMDS) (**E**). Levels of significance determined using analysis of variance (ANOVA) as follows: * *p* < 0.05; ** *p* < 0.01. The treatment group supplied the 0.1% enzyme mixture (*v*/*v*, 2,500,000 Units/kg of arazyme, 200,000 Units/kg of xylanase, and 200,000 Units/kg of mannanase).

In this study, the number of reads per sample was normalized to 10,000 for downstream analyses, preserving greater than 99% coverage (Figure S1A). Alpha-diversity analysis showed that Ace and Chao indices, which indicate species richness, were significantly higher in the treatment group than in the control group (*p* < 0.01) (Figure 1A,B). However, in the case of species evenness, the InvSimpson diversity index showed no significant difference between the treatment and control group. In contrast, the Shannon diversity index showed a significant difference between the groups (*p* < 0.05) (Figure 1C,D). Beta-diversity assessment (tree dendrograms, NMDS, and AMOVA) showed that the treatment group gut microbiota differed significantly from that of the control group (Figure 1E and Figure S1B). These results suggested that feed additive supplementation can significantly alter the gut microbiota of pigs.

**Figure 2:**
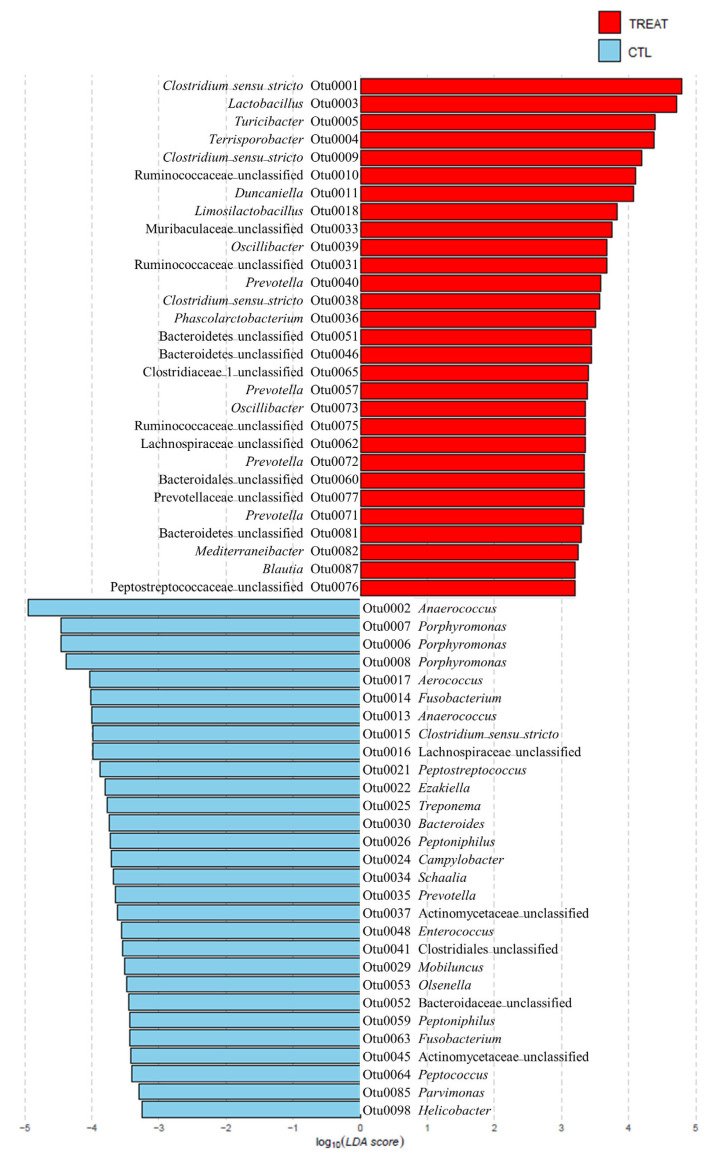
Significantly different relative abundance in operational taxonomic units (OTUs) between the control and treatment groups. The relative abundance was examined using the linear discriminant analysis effect size (LEfSe) (*p* < 0.05). The treatment group supplied the 0.1% enzyme mixture (*v*/*v*, 2,500,000 Units/kg of arazyme, 200,000 Units/kg of xylanase, and 200,000 Units/kg of mannanase).

On checking the taxonomic composition of gut microbiota at the phylum, family, and genus level, the most abundant phyla were Bacteroidetes and Firmicutes. The treatment increased Firmicutes and reduced Bacteroidetes abundance (Figure S2A). The increase in Firmicutes was mainly due to the increase in the family of Ruminococcaceae and the *Lactobacillus*, *Clostridium*_*sensu*_*stricto*, *Terrisporobacter*, and *Turicibacter* genera. The decrease in Bacteroidetes was mainly due to the decrease in the genus *Porphyromonas* (Figure 2, Figure S2B,C). To explain the changes in the gut microbiota due to treatment at a lower taxonomic genus level, we investigated significantly different OTUs through LEFSe. As shown in Figure 2, feed additive consumption significantly increased the abundance of 11 genera (3 *Clostridium*_*sensu*_*stricto*, *Lactobacillus*, *Turicibacter*, *Terrisporobacter*, *Duncaniella*, *Limosilactobacillus*, 2 *Oscillibacter*, 4 *Prevotella*, *Phascolarctobacterium*, *Mediterraneibacter*, and *Blautia*) and 6 unknown genera of the families, 3 Ruminococcaceae, Muribaculaceae, Clostridiaceae, Lachnospiraceae, Prevotellaceae, and Peptostreptococcaceae, and 1 unknown genera of the order, 3 Bacteroidales and 1 unknown genera of the phylum, Bacteroidetes, while decreasing 19 genera (*Anaerococcus*, 3 *Porphyromonas*, *Aerococcus*, 2 *Fusobacterium*, *Anaerococcus*, *Clostridium*_*sensu*_*stricto*, *Peptostreptococcus*, *Ezakiella*, *Treponema*, *Bacteroides*, two *Peptoniphilus*, *Campylobacter*, *Schaalia*, *Prevotella*, *Enterococcus*, *Mobiluncus*, *Olsenella*, *Peptococcus*, *Parvimonas*, and *Helicobacter*) and 1 unknown genera of the order, Clostridiales, and 3 unknown genera of the families, 2 Lachnospiraceae, Actinomycetaceae, and Bacteroidaceae. Among the OTUs that were significantly different between groups, there were 4 OTUs with the same taxonomy (2 *Clostridium*_*sensu*_*stricto*, *Prevotella*, Lachnospiraceae).

### 3.6. PICRUSt Analysis

**Figure 3:**
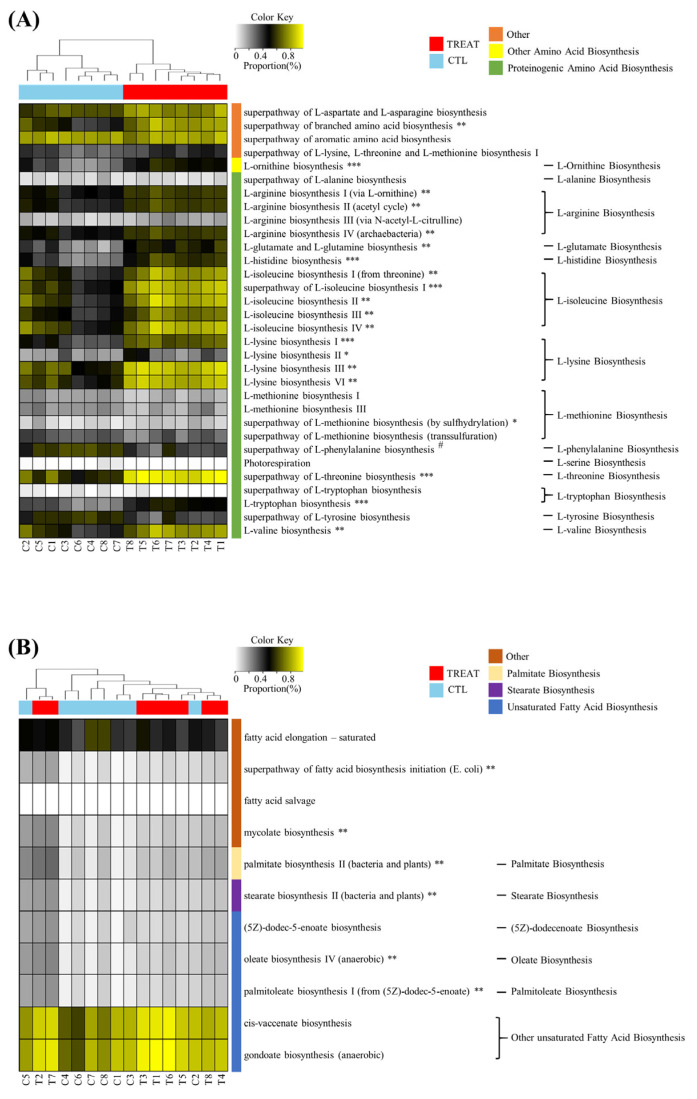
Heatmap analysis of the predicted metabolic pathway. Amino acid biosynthesis (**A**) and fatty acid biosynthesis (**B**). * and # indicate significant differences between the treatment and control groups using ALDEx2 (* *p* < 0.05; # *p* < 0.05; ** *p* < 0.01; *** *p* < 0.001). The treatment group supplied the 0.1% enzyme mixture (*v*/*v*, 2,500,000 Units/kg of arazyme, 200,000 Units/kg of xylanase, and 200,000 Units/kg of mannanase).

The results in Figure 3 show that treatment affected the predicted metabolic pathways of amino acid biosynthesis and fatty acid biosynthesis. In the amino acid biosynthesis pathway, a total of 32 pathways were detected. Of these, 20 predicted metabolic pathways (biosynthesis of branched amino acid, ornithine, arginine, glutamate, histidine, isoleucine, lysine, methionine, threonine, tryptophan, and valine) were significantly increased by the treatment, where most of them were included in proteinogenic amino acid biosynthesis. On the other hand, only one predicted metabolic pathway (phenylalanine biosynthesis) was significantly increased in the CTL group. In the case of fatty acid biosynthesis, a total of 11 predicted metabolic pathways were detected, of which 6 predicted metabolic pathways (fatty acid biosynthesis initiation and biosynthesis of mycolate, palmitate, stearate, oleate, and palmitoleate) were significantly increased by treatment.

**Figure 4:**
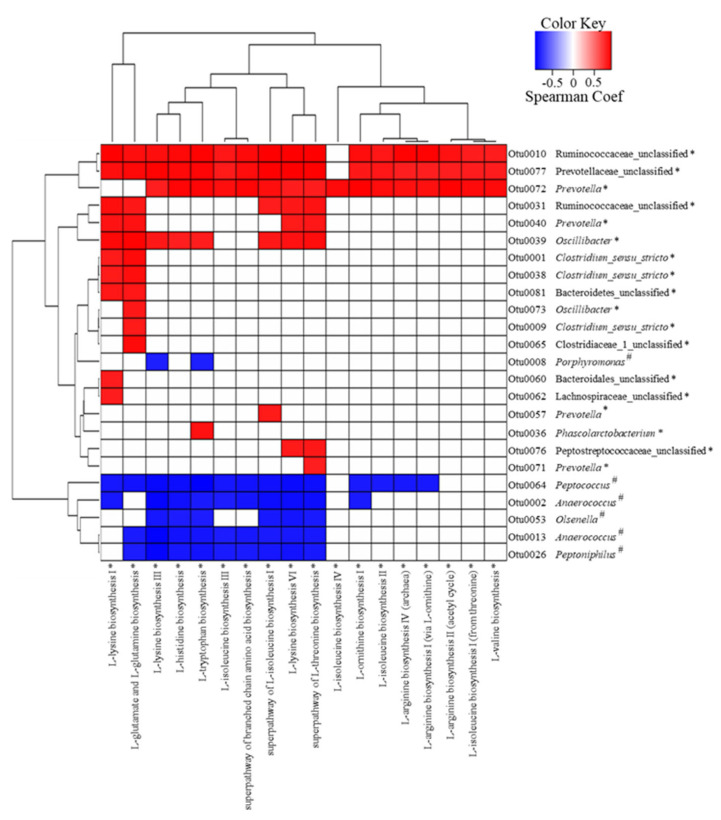
The correlation analysis of LEfSe-selected operational taxonomic units (OTUs) associated with the ALDEx2-selected predicted metabolic pathway by using Spearman’s rank (*p* < 0.01, positive correlation: Spearman Coef > 0.8; negative correlation: Spearman Coef < 0.8). * and # indicate significant differences between the treatment and control groups determined by LEfSe or ALDEx2. The treatment group supplied the 0.1% enzyme mixture (*v*/*v*, 2,500,000 Units/kg of arazyme, 200,000 Units/kg of xylanase, and 200,000 Units/kg of mannanase).

We conducted association analyses of LEfSe-selected OTUs (58 OTUs) with ALDEx2-selected predicted metabolic pathways (32 predicted metabolic pathways) using Spearman’s correlation rank (Figure 4). The results confirmed that 24 OTUs and 18 predicted amino acid biosynthesis pathways were strongly associated, and most pathways were related to EAA. However, no OTU was strongly associated with the predicted fatty acid biosynthesis pathways. Additionally, 3 out of 18 OTUs increased by treatment group (Otu0010: Ruminococcaceae, Otu0077: Prevotellaceae, and Otu00072: *Prevotella*) were positively associated with most of the predicted amino acid biosynthesis pathways, whereas the other 3 OTUs (Otu0031: Ruminococcaceae, Otu0040: *Prevotella*, and Otu0039: *Oscillibacter*) were positively associated with 4 to 8 of the predicted amino acid biosynthesis pathways. The remaining OTUs were positively associated with only 1 or 2 of the predicted amino acid biosynthesis pathways. Furthermore, in the case of 6 OTUs decreased by the treatment, 1 OTU (Otu0064: *Peptococcus*) was negatively associated with most of the predicted amino acid biosynthesis pathways; 4 OTUs (Otu0002: *Anaerococcus*, Otu0053: *Olsenella*, Otu0013: *Anaerococcus*, and Otu0026: *Peptoniphilus*) were negatively associated with a minimum of 6 and a maximum of 10; and 1 OTU (Otu0008: *Porphyromonas*) was negatively associated with L-lysine and L-tryptophan biosynthesis.

## 4. Discussion

Various gut microbes of invertebrates secrete many hydrolytic enzymes and could be new sources of biotechnological applications [5]. The metalloprotease arazyme used in this study was produced by *Serratia proteamaculans* HY-3, a symbiotic bacterium of the spider *Nephila clavata* that can degrade a wide range of proteins and has anti-inflammatory effects in both cell and animal models of atopic dermatitis [7,16]. Additionally, mannanase and xylanase were purified from *Cellulosimicrobium* sp. HY-13, a gut bacterium of *Eisenia fetida* [8] and *Paenibacillus* sp. HY-8, a gut bacterium of *Moechotypa diphysis* [9], respectively. This study used invertebrate symbiotic microbe-derived enzymes with high industrial potential and an enzyme mixture containing arazyme as the main component and xylanase and mannanase as synergistic enzymes as a feed additive to evaluate the growth and meat quality of pigs.

Supplementation of exogenous enzymes in a diet, particularly protease as a part of an enzyme mixture, is widely used with the expectation that it will improve the efficiency of nutrient utilization by neutralizing anti-nutritional factors and increasing nutrient digestibility, thereby, improving growth performance [17]. In our study, dietary supplementation of arazyme, in combination with xylanase and mannanase, affected the final body weight, average slaughter age, ADG, and gain:feed ratio. However, it showed no significant differences in ADFI compared to that of the control group. Other studies also showed that mixtures of enzymes with protease positively affected nutrient digestibility, bacterial populations in the large intestine, and growth performance of pigs [1,18]. Microbial exogenous enzymes hydrolyze dietary components in the small intestine into compounds that can be absorbed. Therefore, improved growth performance due to dietary supplementation of exogenous enzymes may enhance the disruption of the dietary cell walls and increase the exposure of the trapped nutrients, leading to growth-promoting effects. Meat quality is one of the most important economic characteristics of pigs, and it determines the suitability of meat for further processing and storage [19]. Here, the supplementation of arazyme in combination with xylanase and mannanase influenced meat color, cooking loss, shear force, and antioxidant capacity. In contrast, there was no significant effect on pH, drip loss, and TBARS. An earlier study showed that the supplementation of the multi-enzyme mixture (NSPase: 1,4-*β*-xylanase 20,000 U/g; *α*-amylase 2000 U/ g; Protease 40,000 U/g) influenced the cooking loss and color lightness of breast meat in broilers fed a low-metabolizable energy diet [20]. However, the influence of dietary supplementation of multi-enzyme protease as the main component and xylanase and mannanase as synergistic components on meat quality has not been previously reported. Therefore, comparisons could not be made with other studies. In the present study, enzyme supplementation changed the free amino acid profiles. The free amino acid content was 0.476% for the treatment group, which was greater than that of the control group (0.404%). In addition, the treatment group had high levels of lysine, valine, leucine, arginine, and isoleucine. These results infer that the meat quality will be excellent as the treatment group shows improved protein composition compared to the control group. Recent studies have shown that certain amino acids directly participate in taste and could also promote protein synthesis and skeletal muscle growth by activating major signaling pathways [21,22]. Therefore, these results suggest that supplementing arazyme in combination with xylanase and mannanase could improve the growth and value of meat in growing–finishing pigs. Additionally, our results showed that C18:1n-9, C16:0, C18:0, and C18:2n-6 were the most abundant fatty acids in the longissimus muscle; thus, the results correlated well with those in earlier studies [23,24]. Furthermore, our study revealed higher MUFAs and lower SFAs levels in the treatment group compared to that of the control group. A lower composition of SFAs is considered beneficial from a dietary perspective as SFAs (C14:0 and C16:0) are associated with a cholesterol elevating effect [25]. However, the experimental evidence of exogenous enzymes derived from invertebrate symbionts and their appropriate proportions for improving host and meat quality is limited. Further studies investigating the correlation between exogenous enzyme additives and growth performance, meat quality, and gut microbiota studies are needed to confirm the results from this study.

In this study, feed additive supplementation increased the family Ruminococcaceae and the genera *Lactobacillus* and *Limosilactobacillus* (formerly known as *Lactobacillus*). This is consistent with earlier reports of changes in the gut microbiome when a single enzyme or similar multi-enzyme was added to feed in livestock [26,27,28]. The diet and its utilization in the intestine play an important role in preserving the diversity of gut microbiota; we conjecture that supplementation of exogenous enzymes promotes nutrient utilization and, thus, more diverse microbiota in the intestine. An earlier study showed that *Lactobacillus reuteri* 1, belonging to *Lactobacillus*, supplemented into the pig diet improved the meat quality by altering the muscle fiber characteristics and increasing flavor substances such as glutamic acid [29]. Feed additive consumption also increased *Turicibacter* and *Oscillibacter*. *Turicibacter* is a bacterium that ferments organic compounds and is reported to play a positive role in the microbiome immune interaction and promote the growth performance of pigs [30]. It was reported that *Oscillibacter* was abundant in the feces of pigs with high meat quality compared with those of pigs with low meat quality [31]. Furthermore, treatment consumption increased the abundance of short-chain fatty acids (SCFAs), producing bacteria such as Ruminococcaceae, Muribaculaceae, *Phascolarctobacterium*, *Mediterraneibacter*, and *Blautia*, but decreased the abundance of potential pathogenic bacteria such as *Porphyromonas*, *Aerococcus*, *Fusobacterium*, *Campylobacter*, and *Helicobacter*. Short-chain fatty acids are a common product of fiber breakdown and affect the energy supply, gut health, and metabolic homeostasis and sustain the electrolyte balance that plays an important role in animal growth performance and meat quality [32]. Furthermore, feed additives can increase or decrease the abundance of other members of the OTUs belonging to Lachnospiraceae, *Prevotella*, and *Clostridium*_*sensu*_*stricto* taxa. Earlier studies reported that *Prevotella* and Lachnospiraceae are SCFAs-producing bacteria [33]. Ingestion of *Clostridium butyricum*, belonging to *Clostridium*_*sensu*_*stricto*, was reported to enhance the growth and meat quality of pigs by enhancing the modulation of the host metabolism and intestinal development [34]. *Clostridium* improves meat quality by modulating serum lipid metabolism, amino acid, and fatty acid composition [35]. Due to the lack of genetic information on these OTUs, additional studies at genus or species level are necessary to investigate their precise roles.

Our data revealed that the feed additive significantly improved the biosynthesis of several predicted amino acids. The feed additive enhanced amino acid production that significantly affected the meat quality and growth performance of pigs [36]. Furthermore, we conducted association analyses of LEfSe-selected OTUs with ALDEx2-selected predicted metabolic pathways. Results revealed that Otu0010 (Ruminococcaceae), Otu0077 (Prevotellaceae), and Otu0031 (*Prevotella*) were increased by the feed additive and had a strong positive correlation with the biosynthesis of most amino acids. It was reported that *Prevotella* (belonging to Prevotellaceae) and *Oscillibacter* (belonging to Ruminococcaceae) positively correlate with amino acids biosynthesis [37]. Furthermore, it was reported that the predicted amino acids, which are positively correlated with the increased microorganisms, have a beneficial effect on meat quality and the growth performance of pigs [38]. It was also reported that lysine, arginine, and glutamic acid increase intramuscular fat, an important factor for meat quality [39]. Threonine helps improve immunity and feed intake [40], and tryptophan stimulates serotonin secretion to reduce stress and improves meat quality [41]. In addition, the biosynthesis of fatty acid also increased by feed additive. Fatty acids can be produced by microorganisms and affect meat quality [42]. It was reported that the influence on flavor and muscle color could be understood by determining the firmness/oiliness of adipose tissue and oxidative stability of muscles [43]. Further meta-transcriptomic, metabolomic, and proteomic studies of the intestinal microbes in pigs are necessary to confirm the predicted metabolic pathways identified in this study. The major factors affecting the health of pigs and gut microbial communities, including the type of diets and dose of enzyme and the species and age of the animal, were limited in this study. Further research is required on the application of exogenous enzymes, particularly metalloprotease, from invertebrate symbiotic bacteria for feed supplementation.

## 5. Conclusions

The supplementation of arazyme in combination with xylanase and mannanase improved the growth performance, meat quality, and gut microbiota. Summarily, the results suggested the application of exogenous enzymes derived from an invertebrate symbiotic bacterium for improving nutrient digestibility and animal performance. This is the first report demonstrating invertebrate symbiotic bacterium-derived exogenous enzymes as a feed additive in the pig industry. Considering the limitations in research related to the exogenous enzymes from an invertebrate symbiotic bacterium (especially metalloprotease), supplementation with growth-enhancing additives, and the meat quality of growing–finishing pigs, the results of this study can be used as a theoretical basis for further studies.

## Figures and Tables

**Table 1:** Ingredient composition of the basal diet for growing–finishing pigs.

Item	Control
Ingredients	
Corn, %	70.25
Soybean meal, %	13.48
Wheat bran, %	5.65
Rapeseed meal, %	2.00
Molasses, %	4.00
Animal fat, %	1.68
Limestone, %	1.00
Tricalcium phosphate, %	0.68
L-Lysine-HCl (78%), %	0.39
DL-Methionine (98%), %	0.14
L-Threonine (98%), %	0.21
L-Tryptophan, %	0.11
Salt, %	0.21
Vitamin premix ^1^, %	0.10
Mineral premix ^2^, %	0.10

^1^ Supplied per kilogram of diet: Vitamin A, 4000 IU; vitamin D_3_, 800 IU; vitamin E, 40 IU; vitamin B_3_, 2 mg; vitamin B_12_, 16 mg; vitamin K, 2 mg; thiamine, 8 mg; riboflavin, 2 mg; pantothenic acid, 11 mg; niacin, 20 mg; biotin, 0.02 mg. ^2^ Supplied per kilogram of diet: CuSO_4_, 130 mg; FeSO_4_, 175 mg; ZnSO_4_, 100 mg; MnSO_4_, 90 mg; CaIO_4_, 0.3 mg; CoNO_3_, 0.5 mg; NaSe, 0.2 mg.

**Table 2:** Chemical composition of the basal diet for growing–finishing pigs.

Item	Control	Feed Additive *
Moisture, %	12.66 ± 0.07	12.66 ± 0.05
Ash, %	4.93 ± 0.08	4.28 ± 0.29
Crude protein, %	11.46 ± 0.94	13.00 ± 1.01
Crude fat, %	10.67 ± 0.97	9.00 ± 0.97
Crude fiber, %	0.18 ± 0.07	0.27 ± 0.13
Carbohydrate, %	60.28 ± 1.76	61.06 ± 2.19
NFE, %	60.11 ± 1.67	60.86 ± 2.22
Total nitrogen, %	1.83 ± 0.15	2.08 ± 0.16
Lysine, mg/kg	1735.69 ± 157.54	2170.24 ± 758.84
Methionine, mg/kg	298.56 ± 39.59	298.21 ± 49.23
Threonine, mg/kg	672.33 ± 34.03	630.85 ± 103.94
Tryptophan, mg/kg	211.12 ± 2.96	223.34 ± 51.08
Isoleucine, mg/kg	111.59 ± 0.36	130.77 ± 23.28
Valine, mg/kg	192.43 ± 1.40	212.94 ± 37.88
Myristic acid (C14:0), %	1.60 ± 0.04	1.56 ± 0.01
Palmitic acid (C16:0), %	19.34 ± 0.16	19.01 ± 0.73
Stearic acid (C18:0), %	7.12 ± 0.31	6.89 ± 0.73
Arachidic acid (C20:0), %	0.16 ± 0.08	0.17 ± 0.24
Lauric acid (C12:0), %	1.48 ± 0.22	1.52 ± 0.02
Behenic acid (C22:0), %	0.13 ± 0.18	0.12 ± 0.17
Lignoceric acid (C24:0), %	0.16 ± 0.22	0.15 ± 0.21
Palmitoleic acid (C16:1n-7), %	1.30 ± 0.02	1.13 ± 0.08
Oleic acid (C18:1n-9), %	32.52 ± 0.82	31.89 ± 0.70
Linoleic acid (C18:2n-6), %	34.02 ± 0.01	35.58 ± 0.92
α-linolenic acid (C18:3n-3), %	1.60 ± 0.08	1.46 ± 0.00
Eicosenoic acid (C20:1n-9), %	0.26 ± 0.37	0.25 ± 0.35
Eicosadienoic acid (C20:2), %	0.10 ± 0.13	0.09 ± 0.12
Erucic acid (C22:1n-9), %	0.21 ± 0.30	0.18 ± 0.26
Digestible energy, Kcal/kg	3830.00 ± 54.60	3772.60 ± 37.92

* The tested feed additive contained 2,500,000 Units/kg of arazyme, 200,000 Units/kg of xylanase, and 200,000 Units/kg of mannanase. Data are presented as the means ± SD (*n* = 3).

**Table 3:** Effects of feed additive on the growth performance and carcass traits of growing–finishing pigs.

Item	Control	Feed Additive *	SEM	*p* Value
Growth performances				
Initial body weight, kg	37.90	37.57	1.26	0.572
Final body weight, kg	111.24	115.73	0.43	<0.001
Mortality, %	2.73	1.17		
Average slaughter age, days	106.50	97.50	0.99	0.008
ADG, kg/d	0.690	0.759	0.009	0.014
ADFI, kg/d	2.355	2.501	0.055	0.364
Feed intake, ton	61.02	51.66		
G:F	0.292	0.303	0.003	0.001

* The tested feed additive contained 2,500,000 Units/kg of arazyme, 200,000 Units/kg of xylanase, and 200,000 Units/kg of mannanase and fed the basal diet supplemented with 0.1% enzyme mixture (*v*/*v*) ad libitum. ADG = average daily gain; ADFI = average daily feed intake; G:F = gain:feed ratio. SEM, standard error of means (*n* = 8).

**Table 4:** Impact of the feed additive on carcass traits and meat quality of longissimus muscle in finishing pigs.

Item	Control	Feed Additive *	SEM	*p* Value
Carcass traits				
Hot carcass weight, kg	85.62	89.29	0.01	<0.001
Backfat thickness, cm	1.913	2.141	0.01	0.001
Meat quality				
pH	5.64	5.65	0.01	0.923
Drip loss, %	9.48	9.74	0.27	0.815
Cooking loss, %	22.76	19.32	0.30	0.004
Shear force, kg/cm^2^	19.12	16.50	0.08	<0.001
L* (lightness)	55.23	55.50	0.38	0.883
a* (redness)	9.46	5.71	0.14	<0.001
b* (yellowness)	16.33	12.60	0.14	<0.001
TBARS, mg/kg	0.22	0.22	0.06	0.932
DPPH radical scavenging activity, %	13.52	23.23	0.11	<0.001
ABTS radical scavenging activity, %	47.31	52.11	0.20	<0.001

* The tested feed additive contained 2,500,000 Units/kg of arazyme, 200,000 Units/kg of xylanase, and 200,000 Units/kg of mannanase and fed the basal diet supplemented with 0.1% enzyme mixture (*v*/*v*) ad libitum. TBARS = thiobarbituric acid reactive substances; DPPH = 1,1-diphenyl-2-picrylhydrazyl; ABTS = 3-ethylbenzothiazoline-6-sulfonic acid. SEM, standard error of means (*n* = 8).

**Table 5:** Impact of the feed additive on the free amino acid composition of longissimus muscle in finishing pigs.

Item	Control	Feed Additive *	SEM	*p* Value
EAA, mg/kg				
Leucine	332.72	397.28	1.91	<0.001
Valine	196.13	229.97	1.07	<0.001
Lysine	155.60	223.28	1.23	<0.001
Arginine	209.60	260.14	1.34	<0.001
Isoleucine	165.06	195.36	0.90	<0.001
Phenylalanine	207.36	244.55	1.11	<0.001
Threonine	194.77	226.18	1.14	<0.001
Methionine	168.45	207.85	0.97	<0.001
Histidine	89.06	102.51	0.66	0.001
Tryptophan	126.75	154.67	0.61	<0.001
NEAA, mg/kg				
Glutamine	742.73	872.23	4.41	<0.001
Glutamic acid	375.93	431.69	2.49	0.001
Alanine	ND *	ND *		
Aspartic acid	67.89	78.82	0.95	0.020
Asparagine	91.30	111.08	0.59	<0.001
Proline	180.56	200.49	1.03	0.001
Glycine	292.40	351.39	1.48	<0.001
Serine	242.25	287.81	1.51	<0.001
Tyrosine	203.47	187.79	0.97	0.012
Total EAAs^1^	1845.50	2241.79	10.97	<0.001
Total NEAAs^2^	2196.53	2518.30	13.43	<0.001
Total AAs^3^	4042.03	4760.09	24.39	<0.001

* The tested feed additive contained 2,500,000 Units/kg of arazyme, 200,000 Units/kg of xylanase, and 200,000 Units/kg of mannanase and fed the basal diet supplemented with 0.1% enzyme mixture (*v*/*v*) ad libitum. EAA = essential amino acids; NEAA = non-essential amino acids. * ND, not determined SEM, standard error of means (*n* = 8).

**Table 6:** Impact of the feed additive on the fatty acid composition of longissimus muscle in finishing pigs.

Item	Control	Feed Additive *	SEM	*p* Value
Myristic acid (C14:0), %	1.60	1.50	0.01	0.005
Palmitic acid (C16:0), %	26.46	25.81	0.04	0.003
Palmitoleic acid (C16:1n-7), %	3.43	3.56	0.01	0.013
Stearic acid (C18:0), %	14.27	13.43	0.03	<0.001
Oleic acid (C18:1n-9), %	40.58	41.28	0.06	0.007
Linoleic acid (C18:2n-6), %	9.52	9.90	0.07	0.043
α-linolenic acid (C18:3n-3), %	0.24	0.23	0	<0.001
Arachidic acid (C20:0), %	0.22	0.23	0	<0.001
Eicosadienoic acid (C20:2), %	0.45	0.48	0.01	0.021
Eicosenoic acid (C20:1n-9), %	0.82	0.83	0.01	0.288
Arachidonic acid (C20:4n-6), %	2.08	2.38	0.03	0.010
Heneicosylic acid (C21:0), %	0.34	0.36	0.01	0.070
SFA ^1^, %	42.89	41.34	0.07	<0.001
MUFA ^2^, %	44.82	45.66	0.06	0.005
PUFA ^3^, %	12.29	13.00	0.10	0.042
UFA ^4^, %	57.11	58.66	0.07	<0.001

* The tested feed additive contained 2,500,000 Units/kg of arazyme, 200,000 Units/kg of xylanase, and 200,000 Units/kg of mannanase and fed the basal diet supplemented with 0.1% enzyme mixture (*v*/*v*) ad libitum. ^1^ SFA = saturated fatty acid, C14:0 +C16:0 + C18:0 + C20:0 + C21:0. ^2^ MUFA = monounsaturated fatty acid, C16:1n-7 + C18:1n-9 + C20:1n-9. ^3^ PUFA = polyunsaturated fatty acid, C18:2n-6 + C18:3n-3+ C20:2 + C20:4n-6. ^4^ UFA = unsaturated fatty acid, C16:1n-7 + C18:1n-9+ C18:2n-6 + C18:3n-3+ C20:1n-9+ C20:2 + C20:4n-6^.^ SEM, standard error of means (*n* = 8).

## Data Availability

The data presented in this study are available on request from the corresponding author.

## Supplementary Materials

The following supporting information can be downloaded at: https://www.mdpi.com/article/10.3390/ani13030423/s1, Figure S1: The comparative analysis of gut microbiota. Comparison of coverage (A) and Tree dendrogram based on Bray–Curtis (B); Figure S2: Comparison of the taxonomic compositions. Phylum (A), family (B), and genus (C). The relative abundance was examined using the linear discriminant analysis effect size (LEfSe.). * and # indicate significant difference between control and treatment by LEfSe (*p* < 0.05).
