# Supplementary figures and images for: Enzyme Feed Additive with Arazyme Improve Growth Performance, Meat Quality, and Gut Microbiome of Pigs
Source: Animals (Basel). 2023 Jan 26;13(3):423. doi: 10.3390/ani13030423 (PMC9913082; doi:10.3390/ani13030423)

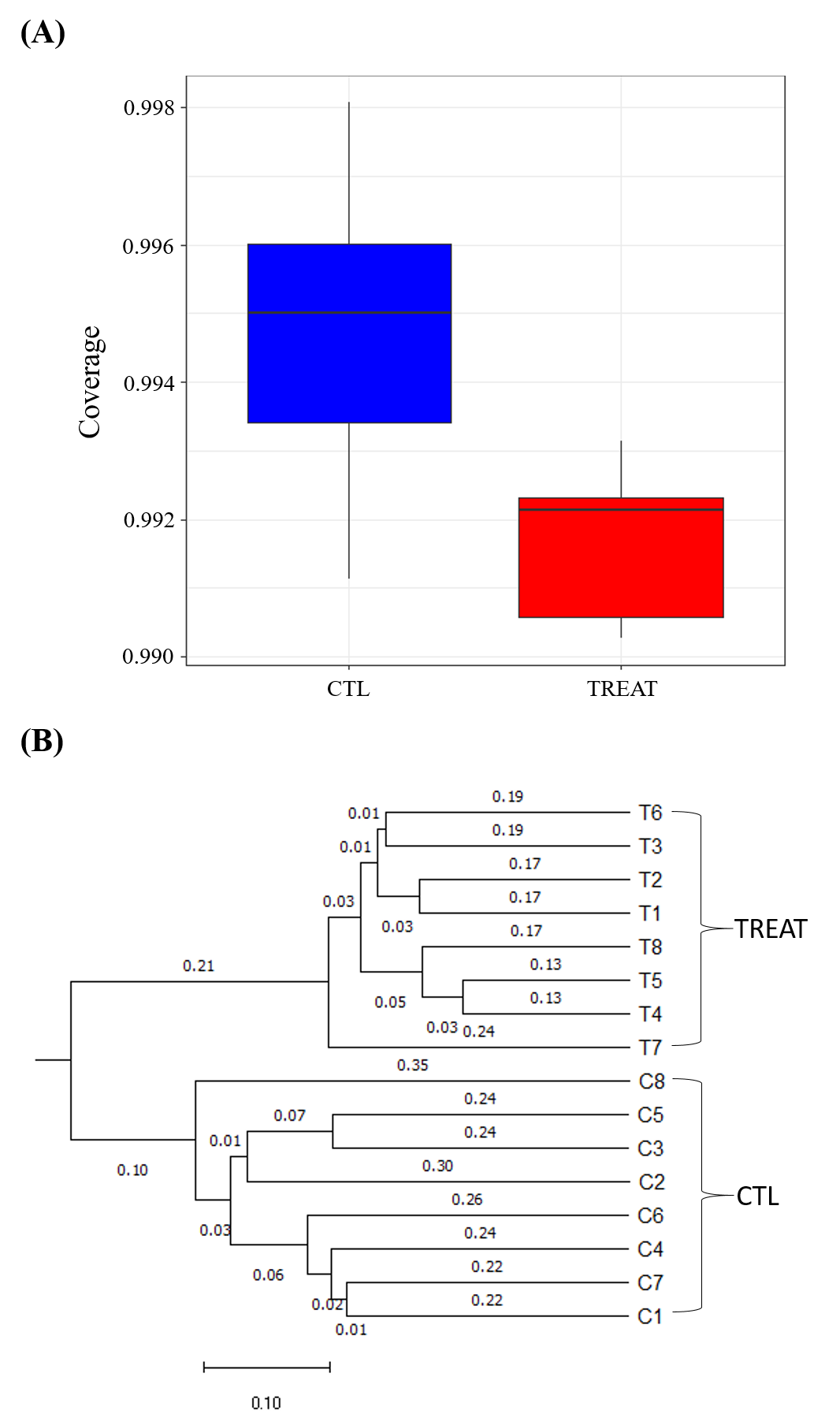

Supplement: Supplementary file 1 [file animals-13-00423-s001.zip › Supplementary Figure S1.tif]

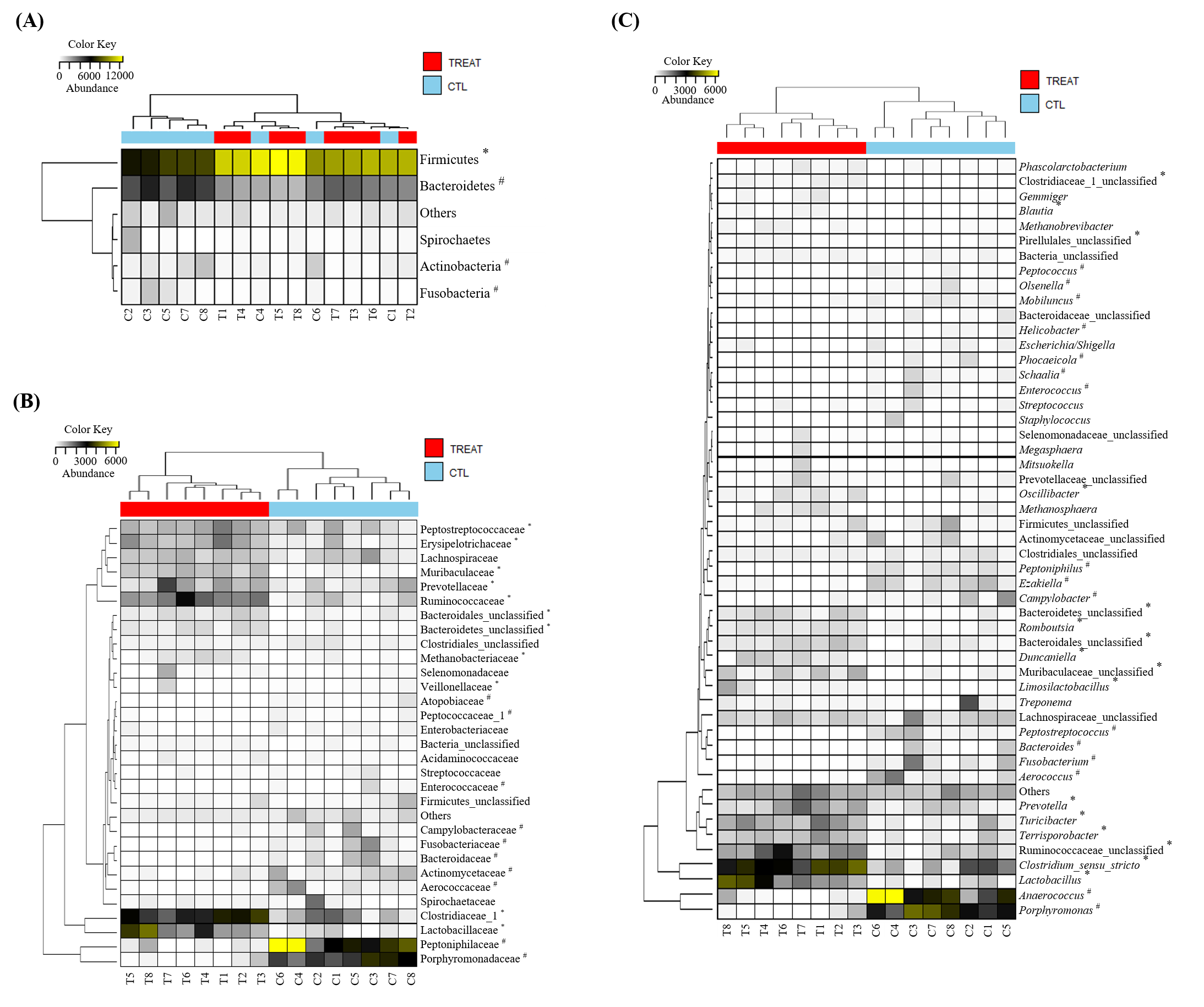

Supplement: Supplementary file 1 [file animals-13-00423-s001.zip › Supplementary Figure S2.tif]
